# THE PROMISE OF FITNESS, HEALTH, AND DIGNITY FOR OLDER ADULTS: A SHOWCASE OF EARLY CAREER RESEARCH IN THE OAIC NETWORK

**DOI:** 10.1093/geroni/igae098.0127

**Published:** 2024-12-31

**Authors:** Karen Bandeen-Roche

**Affiliations:** Chair

## Abstract

The NIH-funded Older Americans Independence Centers (OAICs) aim to produce scientific knowledge allowing older adults to maintain or restore their independence. This national network has a key mission to develop the next generation of investigators and leaders in aging research. Individual OAICs achieve this mission through pilot funding and career development grants, activities and mentorship. The OAIC Coordinating Center additionally provides funding, programming, and an Early Career Faculty Working Group dedicated to fostering emerging scholars. This proposed first-annual GSA showcase features three OAIC-affiliated early career faculty nominated by the Early Career Faculty Working Group based on their receipt of national multi-center pilot awards or invited presentation of their work at monthly OAIC Directors virtual meetings or at the OAIC National Meeting. The talks selected for this symposium illustrate the highly interdisciplinary nature of the OAICs. Juan Pablo Palavicini (UTHSA) will present cross-species pilot work with Andrew Murton (UTMB) to evaluate whether myriocin may inhibit ceramide synthesis, and ultimately preserve muscle and insulin sensitivity in obese older adults. Emma Barber (Northwestern) will present work demonstrating association of heart and respiratory parameters with frailty—and of preoperative physical activity with post-operative complications—in older gynecologic oncology patients, laying groundwork for interventions. Cameron Gettel (Yale) will present findings documenting substantial emergency department use and expenditures by older adults in the last 180 days of life, using Medicare Current Beneficiary Survey data. The symposium concludes with a panel and open discussion addressing aging research career and science topics.

